# Sjogren’s syndrome: Review of the aetiology, Pathophysiology & Potential therapeutic interventions

**DOI:** 10.4317/jced.53605

**Published:** 2017-04-01

**Authors:** Jisha J. Nair, Tejas P. Singh

**Affiliations:** 1 College of Medicine and Dentistry, James Cook University, Townsville, Australia

## Abstract

**Background:**

Sjogren’s syndrome (SS) is an autoimmune disorder characterised by lymphocytic infiltration of exocrine glands, resulting in glandular dysfunction. Objectives: This study aims to review the aetiology of Sjogren’s syndrome, highlight aspects that contribute to the pathophysiology of the disease and explore treatment options that target different mediators of pathogenesis.

**Material and Methods:**

The MEDLINE/PubMed and Google Scholar databases were searched systematically with the terms “Sjogren’s syndrome”; “clinical”; “treatment”; “management”. Eligible studies had to meet a predefined inclusion criteria.

**Results:**

912 identified studies were evaluated against the inclusion criteria. 25 eligible studies were included for review. Sjogren’s syndrome is a multifactorial condition with genetic, environmental and hormonal factors playing a role in establishing the condition. B-cell activating factor (BAFF) is an important mediator in the induction and perpetuation of this condition. Elevated BAFF levels, found in patients with SS, promote growth of B-cells and subsequent production of autoantibody; anti-SSA/Ro. BAFF inhibitors are important potential therapeutic drugs that may be effective in patients with Sjogren’s syndrome. Other potential targets include CD20 and CD22 that cause B-cell depletion.

**Conclusions:**

The pathophysiology of this exocrinopathy has not fully been elucidated. Potential therapeutic interventions include BAFF inhibitors and anti-CD20 and anti-CD22 therapy. However, no clinical trials have been conducted on subjects with Sjogren’s syndrome to support existing research.

** Key words:**Sjogren’s syndrome, autoimmune, rheumatology.

## Introduction

Sjogren’s syndrome (SS) is an autoimmune disorder caused by the lymphocytic infiltration of exocrine glands resulting in glandular dysfunction, preferentially of the salivary and lacrimal glands (1). It can be classified into two types, namely primary Sjogren’s syndrome and secondary Sjogren’s syndrome. Primary Sjogren’s syndrome (pSS) occurs in the absence of other autoimmune diseases and is characterised by keratoconjunctiva sicca (dry eyes) and xerostomia (dry mouth), collectively called the sicca syndrome. In contrast, secondary Sjogren’s syndrome presents along with other autoimmune diseases such as rheumatoid arthritis (RA) and systemic lupus erythematosus (SLE) (2). The prevalence of SS is estimated to be approximately 3% in subjects 50 years or older, with a female to male ratio of 9:1 (3). Conditions associated with SS include rheumatoid arthritis, lupus erythematosus (4) and scleroderma (5). The clinical manifestations are often vague and mistakenly interpreted and attributed to other medical conditions or iatrogenic disorders. As such, incorrect diagnosis of SS is common and approximately half of all patients are thought to be undiagnosed (6). This study aims to review the aetiology of Sjogren’s syndrome, highlight aspects that contribute to the pathophysiology of the disease and explore treatment options that target different mediators of pathogenesis.

## Material and Methods

-Protocol 

This systematic review was conducted with reference to the Preferred Reporting Items for Systematic reviews and Meta-Analyses (PRISMA) guidelines (7). The review focused on studies which highlighted aetiological and pathological components of the disease, as well as potential therapeutic targets and interventions.

-Eligibility Criteria 

All published data on Sjogren’s syndrome from 1980 onwards were searched. To be eligible, studies had to have a focus on SS with regards to at least one of the following: clinical manifestations, pathophysiology and treatment. Case reports, reviews, editorials and letters were excluded. No restrictions were placed in regards to study design as literature on SS is limited (8). Furthermore, the intention of this study was to provide a holistic overview on this subject.

-Search Strategy 

The following search strategy was employed. Firstly, the MEDLINE/PubMed (US National Library of Medicine, MD, USA) and Google Scholar (Google Mountain View, CA, USA) database were searched. The following terms were used: ‘Sjogren’s syndrome’; ‘clinical’; ‘aetiology’; ‘pathophysiology’; ‘treatment’; ‘management’. Hand searching of references and the use of the related articles on PubMed were performed to identify any additional studies.

## Results

-Search results 

**Figure 1:**
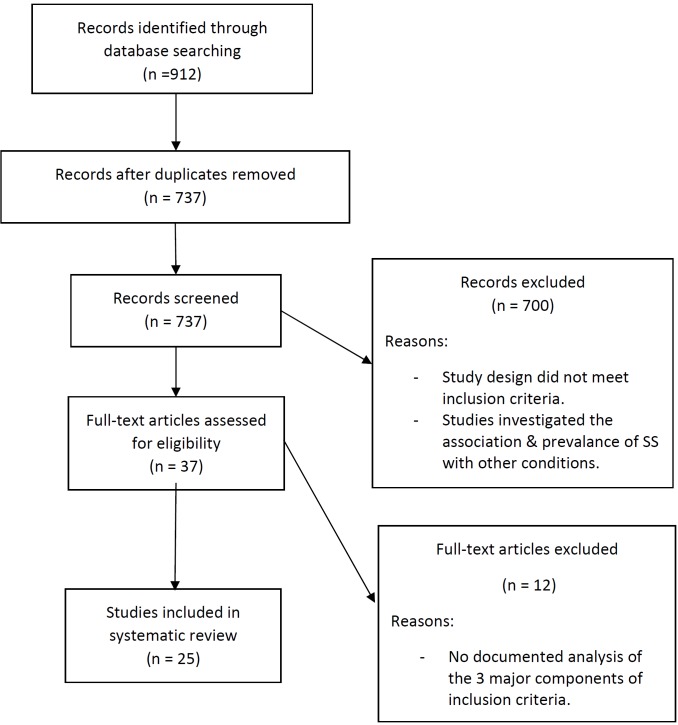
Study design.

855 studies were identified through database searching and a further 57 studies were obtained through hand searching of references. 175 duplicate studies were discarded. The remaining 737 studies were screened on the basis of their abstract/title. 700 full-text articles that did not meet the inclusion criteria were excluded. These included reviews (9,10) and studies that investigated the association and prevalence of other conditions (11) in patients with SS. The remaining 37 articles were evaluated against the eligibility criteria. Finally, 25 remaining studies were included in this study (Fig. 1). Any inconsistencies regarding data were resolved by discussion between the authors.

-Aetiology

•Genetic predisposition 

Genetic predisposition to Sjogren’s syndrome can be attributed to the alleles within the major histocompatibility complex (MHC) class II gene region, in particular HLA-DR and HLA-DQ alleles. These gene associations vary according to ethnic backgrounds of patients. In Californian Caucasian individuals with pSS, the haplotype HLA-DRB1*0301-DRB3*0101-DQA1*0501-DQB1*0201 was found to be associated with the development of the condition (12). Among Japanese women with autoantibodies found in patients with SS, there was an increased frequency of the HLA class II haplotype DRB1*08032/DQA1*0103/DQB1*0601 and DRB1*08032 alleles compared to normal controls (13). However, in another study, this haplotype was predominantly in Chinese patients while Japanese patients had an increased frequency of the haplotype HLA-DRB1*0405-DRB4*0101-DQA1*0301-DQB1*0401 (12). Further study in this area is warranted to establish a definitive link.

•Environmental Factors 

Environmental factors including infectious agents, particularly viruses, are considered to be involved in the pathogenesis of Sjogren’s syndrome. It is thought that viruses promote autoantibody production through means of molecular mimicry resulting in tissue destruction (14). Chronic Hepatitis C virus (HCV) has been implicated in the development of SS in a subset of patients (8). Epstein - Barr virus (EBV) may also have a role in the B lymphoproliferation of the lacrimal gland that occurs in Sjogren’s syndrome (15) Human T-cell leukaemia virus 1 (HTLV-1) also seems to be associated with the condition as mice with the HTLV-1 tax gene have been reported to develop exocrinopathy resembling that of Sjogren’s Syndrome (16).

•Hormonal Factors

The role of oestrogen deficiency can be used to explain the predominance of Sjogren’s syndrome in women compared to men. Animal studies show a correlation between decreased oestrogen levels and SS-like symptoms. Mice with inactivated aromatase genes, and hence the inability to synthesise oestrogen, developed signs of autoimmunity which resemble Sjogren’s syndrome (17) Another study conducted on mice suggests that transgenic (Tg) expression of the retinoblastoma-associated protein 48 (RbAp48) induces tissue specific apoptosis in exocrine glands; dependant on the level of estrogen defiency (18). However, studies on humans have shown contrasting results (19-21). Oestrogen receptor agonists may have potential in the prevention and treatment of autoimmune exocrinopathies such as Sjogren’s syndrome. Futhermore, prolactin is a pro-inflammatory hormone that promotes oestrogen activity, which at high levels, inhibits estrogen production (22).

•Pathophysiology

The mechanism by which Sjogren’s syndrome develops, although widely researched, is not clear. As there is no sequential order in the development of this disease and numerous factors seem to contribute to the progression of the condition, this section will review the pathophysiology focusing on the role of epithelial cells, cytokines, T-lymphocytes and B-cell activating factor (BAFF) in the pathology of SS as well as the autoantibodies secreted by B lymphocytes.

Essentially, in the presence of a genetic predisposition, and an environmental or hormonal trigger, glandular epithelial cells become deregulated and release chemokines and adhesion molecules. This triggers migration of dendritic cells and lymphocytes (T cells and B cells) to the glands. Dendritic cells in the glands produce high levels of interferons (IFN), which causes further retention of lymphocytes in the tissues and their subsequent activation. Interferons stimulate the production of BAFF by epithelial cells, dendritic cells and T cells. BAFF promotes irregular B-cell maturation, leading to the formation of autoimmune B-cells, which secrete autoantibodies locally (23).

•Role of Epithelial Cells

Epithelial cells of salivary and lacrimal glands appear to play an important role in the induction and perpetuation of inflammatory processes in patients with primary Sjogren’s syndrome. Salivary glands, although not classified as antigen presenting cells, appear to express features required to function as one. Upregulation of adhesion molecule CD54/ICAM-1 as well as CD40 and MHC-I on the epithelial cells, and presence of functional TLR2, -3, and -4 molecules on these cells allow them to act as antigen presenting cells (24). This suggests the intrinsic activation of the epithelial cells in Sjogren’s syndrome and support their role in the pathophysiology of the disorder.

•Role of Dendritic Cells and Interferon-alpha (IFN-α)

Plasmacytoid dendritic cells (pDC) and interferon-alpha (IFN-α) appear to influence the pathogenesis of Sjogren’s syndrome. Enhanced levels of pDC and an upregulation of IFN-inducible genes are detected in salivary glands of patients with SS. Plasmacytoid dendritic cells are recruited from the blood to the salivary glands where they secrete increased amounts of IFN-α. This in turn causes the abnormal retention of lymphocytes in the gland tissues and subsequent activation of these lymphocytes (25,26).

•Role of T-lymphocytes

Glandular infiltration of salivary and lacrimal glands causes epithelial destruction, one of the major immunopathologic events, that subsequently results in the replacement of the gland tissue by mononuclear cells. CD4+ T lymphocytes comprise majority of the glandular infiltration in primary Sjogren’s syndrome. These cells destroy the epithelial cells using two major pathways: apoptosis and perforin-granzyme secretion by cytotoxic T cell population (26). The cytotoxic activity of activated T cells could be associated with the expression of CD11c molecule on majority of the T lymphocytes in SS lesions. These molecules, usually present on monocytes, macrophages, and B lymphocytes, may be the factor enabling these cells to adhere to epithelial cells and induce cytotoxic activity (27).

•Role of Cytokines

Cytokines secreted by T-cells infiltrating the glands play a role in the development of the disease. These cytokines include TH1 cytokines such as interferon-gamma (IFN-γ) and interleukin-2 (IL-2) as well as interleukin-10 (IL-10), interleukin-6 (IL-6) and tumour growth factor-beta (TGF-β) (28). However, literature regarding the specific type of cytokine involved in this process has generated conflicted results. A study conducted by Mitsias and colleagues. found that the immune responses are influenced by the balance between type-1 and type-2 cytokines. Cytokines produced by TH2 helper cells dominate in the early lesions of Sjogren’s syndrome, while TH1 helper cells cytokines are associated with later stages of the disorder (29).

•Role of B-cell activating factor (BAFF)

B-cell activating factor (BAFF) is an important mediator involved in the initiation and perpetuation of B-cell dysregulation, commonly seen in Sjogren’s syndrome (30). Secreted by salivary epithelial cells, T cells, and also B-cells, sera of patients with SS have elevated levels of circulating BAFF compared with levels in healthy individuals (31).

Expression of BAFF in secondary lymphoid tissue is essential for survival of mature B-cells. BAFF also has a role in inducing apoptosis in self-reactive B-cells at distinct checkpoints during B-cell maturation. Autoreactive cells have reduced responsiveness to BAFF and therefore, do not survive due to limited levels of BAFF *in vivo* (32). However, overexpression of BAFF, in mice, is shown to be associated with production of autoantibodies and ectopic germinal centre formation (33,34). Studies demonstrate that elevated BAFF levels subvert B-cell self-tolerance by saving the auto-reactive B-cells from underdoing apoptosis. It amplifies B-cell signalling and promote their maturation and differentiation into plasma cells that secrete autoantibodies (31,35). Therefore, BAFF production may be an important event in the autoimmunity.

Plasma BAFF levels in SS patients is associated with elevated presence of autoantibodies, including anti-SSA/SSB. This suggests its role in activating auto-reactive B-cells and modulating the level of autoantibody production (36). This has therapeutic implications in patients as anti-BAFF antibodies or BAFF antagonists may be used to treat Sjogren’s syndrome.

BAFF inhibitors have therapeutic potential in the management of Sjogren’s syndrome. Belimumab is a human monoclonal anti-body against BAFF that inhibits it’s activity, subsequent B-cell development, and autoantibody production. The efficacy of this drug has been trialled on patients with systemic lupus erythematosus (SLE), another autoimmune disorder with B-cells playing a prominent role in the pathogenesis (37). Although the drug is active *in vivo*, it does not provide substantial evidence for reduction in SLE disease activity. Belimumab has completed a phase I trial in SLE and is currently in a phase II trial for SLE and RA (38). There have been no trials to demonstrate the efficacy of this drug in reducing Sjogren’s syndrome but is a potential therapeutic intervention.

Atacicept is a non-selective BAFF blocker, another possible drug for therapeutic use. It is a TACI-Ig fusion protein that binds to both BAFF and a proliferation-inducing ligand (APRIL) receptors on B lymphocytes and inhibits their stimulation. A study on patients with SLE observed a decrease in B lymphocyte and autoantibody levels; however it did not reach a definitive conclusion regarding effect on disease activity (39). Atacicept is a potential treatment approach that requires trials to determine its effectiveness in patients with Sjogren’s syndrome.

Briobacept (BR3-FC) is a selective BAFF blocker that binds to BAFF but not to APRIL. As a result of binding to BAFF, it blocks BAFF-mediated survival and proliferation of B lymphocytes. Studies on monkeys have demonstrated a reduction in B-cells in the peripheral blood and lymphoid tissue after administration of BR3-FC. In addition, it also decreases marginal zone and follicular B cells in tissues, which seems to be unique to briobacept (40). It, therefore, has potential therapeutic use for Sjogren’s syndrome but requires human trials to demonstrate its efficacy.

•Role of Autoantibodies

Autoantibodies, namely anti-SSA/Ro, present in patients with Sjogren’s syndrome are a characteristic feature of this condition. Anti-SSA/Ro mainly targets the autoantigen Ro52 (41). However, as with many mediators involved in this disease, the precise role of these autoantibodies in the pathogenesis of this condition is not clearly understood.

Ro52 (an interferon-inducible protein) mediates ubiquitination of several interferon regulatory factor (IRF) transcription factor family, and subsequently regulate cytokine production. Ro52 negatively regulates IRF activity and hence, inhibits the production of inflammatory cytokines. In the presence of anti-Ro52 and therefore, in patients with Sjogren’s syndrome, ubiquitination of Ro52 is inhibited by the autoantibody. This leads to increased production of pro-inflammatory cytokines regulated by IRF and hence contributes to the pathogenesis of SS (42).

•Other potential therapeutic intervention

B-cell depletion is a form of treatment that can be used in patients with Sjogren’s syndrome. This is achieved by anti-CD20 or anti-CD22 therapy. Rituximab (anti-CD20) is a monoclonal antibody that targets CD20 antigen found on B-cells. The mechanism of the drug, although not completely understood, is believed to involve complement-dependent cytotoxicity, growth inhibition and apoptosis of B-cells (43) Reduction of B-cells subsequently decrease the amount of autoantibodies produced and hence, decrease the effects of the condition. It has been shown to be effective in improving SS symptoms and increasing salivary gland functions (44).

Immunosuppressive drugs can be used for management of systemic disease manifestations. Methotrexate has been shown to improve subjective symptoms of dry mouth and eyes, while there is no improvement in flow rates of lacrimal and salivary glands (45).

## Discussion

Research conducted to study the pathogenesis of Sjogren’s syndrome, and ones referred to in this review, have mostly been on animals such as mice and rats. This is a major limitation with regards to applying these findings to humans. Future studies should be aimed at developing randomised controlled trails, with large sample sizes and longer follow-up to support these findings in the human population. Most studies highlight a correlation between cell mediators/cells and Sjogren’s syndrome, but have not established causation. In regards to therapeutic trials, most studies have been conducted on a small number of patients with SLE (38) and not on patients with SS. Although widely researched, the precise mechanism of glandular destruction that occurs in SS is not well elucidated. Epithelial cells of the glands, cytokines, T lymphocytes and BAFF have all been demonstrated to contribute to the pathogenesis of this condition.

## Conclusions

This study reviewed the aetiology, pathophysiology and possible therapeutic interventions of Sjogren’s syndrome, an autoimmune disorder characterised by glandular infiltration of exocrine glands. Potential therapeutic interventions include BAFF inhibitors and anti-CD20 and anti-CD22 therapy. However, there have been no therapeutic trials conducted specifically on patients with Sjogren’s syndrome.
